# The Adulteration of Drugs

**Published:** 1899-11-11

**Authors:** 


### THE ADULTERATION OF DRUGS.

An article which appeared in The Hospital on October 21st, page 44, entitled "The Adulteration of Drugs," has given rise to various comments which have not always been fair. Our relations with Dr. Paul we must put on one side. There was a distinct misunderstanding, and for our error in regard to this we have frankly apologised to Dr. Paul. In regard to other matters, however, the case is different. It is distinctly untrue to say as is said in the *Pharmaceutical Journal*, in regard to the report we published, "that The Hospital suppresses data in that report, which, if published, would have shown that the statements made in the article are directly opposite to the real facts of the case, and that the damaging imputations made are not in any way justified." Not a single word in that report has been suppressed except the names of certain firms, and we entirely fail to see how these names, if published, could in any way "have shown that the statements made in the article are directly opposite to the real facts of the case."

The facts of the case are abundantly simple. We collected certain samples of plaisters and forwarded them for analysis. Finding, however. that it would be better to include a larger number, we instructed our manager, who had charge of the matter, who wrote to the gentleman entrusted with the investigations in the following words: "I understand that you do not see your way to making any alteration in your fee. I, therefore, agree to this charge, which will include the purchase of further samples by you, and leave the whole matter in your hands with confidence. I presume you will make a point of collecting samples from every possible source, so that the report will embrace the whole market. . . ." What could be plainer or more fair? The places at which these samples were bought embraced the City, the West-end, and various suburbs. The plaisters so collected were made by 15 different firms, and in more than one instance plaisters by the same firm were collected from the shops of different chemists. These shops included establishments of various kinds, one or two of them being so-called "stores," but others being the establishments of pharmaceutical chemists of the highest class. There the matter stands. We pointed out the wrong done to the public, but we insisted equally on the wrong done to those chemists who supply drugs of standard strength, and in so doing we endeavoured to express the truth. If we could have added the names it would no doubt have added piquancy to the article, but it would have in no way altered its teaching.

